# [^68^Ga]-NODAGA-RGD Positron Emission Tomography (PET) for Assessment of Post Myocardial Infarction Angiogenesis as a Predictor for Left Ventricular Remodeling in Mice after Cardiac Stem Cell Therapy

**DOI:** 10.3390/cells9061358

**Published:** 2020-05-30

**Authors:** Cajetan Immanuel Lang, Piet Döring, Ralf Gäbel, Praveen Vasudevan, Heiko Lemcke, Paula Müller, Jan Stenzel, Tobias Lindner, Markus Joksch, Jens Kurth, Carina Bergner, Hans-Jürgen Wester, Hüseyin Ince, Gustav Steinhoff, Brigitte Vollmar, Robert David, Bernd Joachim Krause

**Affiliations:** 1 Department of Cardiology, Rostock University Medical Center, 18057 Rostock, Germany; Cajetan.Lang@med.uni-rostock.de (C.I.L.); Hueseyin.Ince@med.uni-rostock.de (H.I.); 2 Department of Cardiac Surgery, Rostock University Medical Center, 18057 Rostock, Germany; Piet.Doering@uni-rostock.de (P.D.); Ralf.Gaebel@med.uni-rostock.de (R.G.); Praveen.Vasudevan@med.uni-rostock.de (P.V.); Heiko.Lemcke@med.uni-rostock.de (H.L.); Paula.Mueller@uni-rostock.de (P.M.); Gustav.Steinhoff@med.uni-rostock.de (G.S.); 3 Department of Nuclear Medicine, Rostock University Medical Center, 18057 Rostock, Germany; Jens.Kurth@med.uni-rostock.de (J.K.); Carina.Bergner@med.uni-rostock.de (C.B.); Bernd.Krause@med.uni-rostock.de (B.J.K.); 4 Department of Life, Light and Matter, Rostock, University of Rostock, 18057 Rostock, Germany; 5 Core Facility Multimodal Small Animal Imaging, University Medical Center, 18057 Rostock, Germany; Jan.Stenzel@med.uni-rostock.de (J.S.); Tobias.Lindner@med.uni-rostock.de (T.L.); Markus.Joksch@med.uni-rostock.de (M.J.); 6 Pharmaceutical Radiochemistry, Technische Universität München, 85748 Garching, Germany; H.J.Wester@tum.de; 7 Rudolf-Zenker-Institute for Experimental Surgery, Rostock University Medical Center, 18057 Rostock, Germany; Brigitte.Vollmar@med.uni-rostock.de

**Keywords:** angiogenesis, myocardial infarction, [^68^Ga]-NODAGA-RGD, α_v_β_3_-integrin, cardiac induced cells

## Abstract

Angiogenesis plays a central role in the healing process following acute myocardial infarction. The PET tracer [^68^Ga]-NODAGA-RGD, which is a ligand for the α_v_β_3_ integrin, has been investigated for imaging angiogenesis in the process of healing myocardium in both animal and clinical studies. It’s value as a prognostic marker of functional outcome remains unclear. Therefore, the aim of this work was to establish [^68^Ga]-NODAGA-RGD for imaging angiogenesis in the murine infarct model and evaluate the tracer as a predictor for cardiac remodeling in the context of cardiac stem cell therapy. [^68^Ga]-NODAGA-RGD PET performed seven days after left anterior descending coronary artery (LAD) occlusion in 129S6 mice showed intense tracer accumulation within the infarct region. The specificity was shown in a sub-group of animals by application of the competitive inhibitor cilengitide prior to tracer injection in a subgroup of animals. Myocardial infarction (MI) significantly reduced cardiac function and resulted in pronounced left ventricular remodeling after three weeks, as measured by cardiac MRI in a separate group. Cardiac induced cells (CiC) that were derived from mESC injected intramyocardially in the therapy group significantly improved left ventricular ejection fraction (LVEF). Surprisingly, CiC transplantation resulted in significantly lower tracer accumulation seven days after MI induction. Accordingly, we successfully established the PET tracer [^68^Ga]-NODAGA-RGD for the assessment of α_v_β_3_ integrin expression in the healing process after MI in the mouse model. Yet, our results indicate that the mere extent of angiogenesis following MI does not serve as a sufficient prognostic marker for functional outcome.

## 1. Introduction

Acute myocardial infarction (MI) is a leading cause of death in most developed countries and is mostly induced by the occlusion of a coronary artery. Early revascularization reduces infarct size and improves the prognosis of MI patients [1]. Yet, the remaining damage causes pathological remodeling of the myocardium and it can potentially result in congestive heart failure. Myocardial healing following the ischemic event is a highly dynamic and complex process, which can be divided into three distinct phases [2]. The early inflammatory phase is dominated by infiltrating leucocytes scavenging the wound of necrotic tissue. A wound containing fibroblasts, macrophages, and a rich vascular network is formed at the next stage. Fibroblasts differentiate into myofibroblasts and deposit a provisional extracellular matrix, which is rich in fibrin and fibronectin. This proliferative phase is highly energy demanding and it depends on effective angiogenesis, a process that is defined as sprouting of pre-existing post-capillary venules into the provisional extracellular matrix. Eventually, myofibroblasts undergo apoptosis, microvessels regress, and a collagen-rich mature scar replaces the metabolically active immature scar [2]. 

Angiogenesis, being both an integral and an indispensable part of the wound healing [3], has been shown to be essential for myocardial regeneration after MI [4]. Where the inhibition of angiogenesis promotes heart failure in a murine model of cardiac hypertrophy [5], the induction of angiogenesis by cardiac stem cell therapies has been suggested by many researchers to preserve cardiac function following myocardial infarction in both small and large animals [6]. 

Despite these promising findings of proangiogenic therapeutic approaches in preclinical models, the results from clinical trials have been disappointing. Unfortunately, the reason for this remains unexplained, as only functional effects (such as LVEF and NYHA classification) of novel therapies can be assessed in patients. The underlying suggested biological mechanism—angiogenesis—is not amenable to established clinical diagnostic methods. The urgent need for non-invasive assessment of angiogenesis has inspired the field of molecular imaging to develop novel tracers that target the process of post-infarction angiogenesis. Thereby, the majority of single photon emission tomography (SPECT) and positron emission tomography (PET) tracers have been designed for binding to the α_v_β_3_-integrin via the amino acid sequence RGD [7]. This integrin represents a vitronectin receptor, which is highly expressed at the tips of capillaries sprouting into the provisional matrix of granulation tissue [3,8,9,10]. Accordingly, imaging myocardial angiogenesis following ischemic damage has been successfully established with different tracers for various animal models in the preclinical setting at the level of qualitative and visual assessment of α_v_β_3_-integrin expression [11,12]. 

Surprisingly, in animals, there is only one study correlating focal accumulation of [^18^F]-galacto-RGD with infarct size [12], and another single study correlates the amount of tracer uptake with angiogenesis following stem cell therapy [13]. Furthermore, PET based imaging for the mouse model is not available to date, despite its unique features for basic research.

We have recently introduced a therapeutic approach for myocardial repair that is based on the transplantation of cardiac induced cells (CiC). These cells are derived from murine embryonic stem cells, exhibit an expression profile of cardiac marker genes, beat robustly, and improve cardiac function when transplanted after myocardial infarction in mice. Cell therapy also changed the inflammatory profile, as assessed by [^18^F]-FDG PET [14]. Thus, we hypothesized that CiC transplantation modulates angiogenesis in the process of post-ischemic myocardial healing and chose this therapeutic approach for evaluating [^68^Ga]-NODAGA-RGD PET for imaging angiogenesis and its modulation in a murine model of myocardial infarction.

Therefore the goal of the preset study was to:

1. establish the PET Tracer [^68^Ga]-NODAGA-RGD for imaging angiogenesis in a murine model of myocardial infarction;
2. assess the effect of CiC transplantation on LV-remodeling post infarction measured by cardiac MRI (CMR); and,
3. evaluate whether α_v_β_3_-integrin expression in the infarct, as measured by [^68^Ga]-NODAGA-RGD-PET, predicts cardiac function following cell therapy.

## 2. Materials and Methods

### 2.1. Stem Cell Culture and Cardiovascular Differentiation

In order to avoid immune rejection of the transplanted cells W4 murine embryonic stem cells (mESCs), originally isolated from the 129S6 mouse strain [15], were used for syngeneic transplantation into 129S6/SvEvTac mice. The cells were grown in DMEM supplemented with 15% FBS Superior (Biochrom AG, Berlin, Germany), 1% Cell Shield^®^ (Minerva Biolabs GmbH, Berlin, Germany), 100 µM non-essential amino acids, 1000 U/mL leukemia inhibitory factor (Phoenix Europe GmbH, Germany), and 100 µM β-mercaptoethanol (Sigma–Aldrich GmbH, Taufkirchen, Germany) at 37 °C, 5% CO_2_, and 20% O_2_, according to established protocols in our laboratory [16]. We initiated cardiovascular differentiation by the use of cardiogenic differentiation medium, containing IBM (Iscove’s Basal Medium, Biochrom AG, Berlin, Germany) supplemented with 10% FBS Superior, 1% Cell Shield^®^, 100 µM non-essential amino acids, 450 µm 1-thioglycerol (Sigma–Aldrich GmbH), and 213 µg/mL ascorbic acid (Sigma–Aldrich GmbH), as described previously [16]. Thereby, differentiation was initiated by hanging-drop culture for two days at 37 °C, 5% CO_2_, and 20% O_2_. We started the formation of embryoid bodies (EB) by plating 400 cells per drop on the cover of a square petri dish. After two days of hanging-drop culture, EB were grown for an additional four days in suspension culture [17], and then harvested for transplantation. In the following we will refer to these cells as cardiac induced cells (CiC), which we characterized in detail in a previous work [14].

### 2.2. Animal Model

The federal animal care committee of the Landesamt für Landwirtschaft, Lebensmittelsicherheit und Fischerei Mecklenburg-Vorpommern (LALLF, Rostock, Germany) approved all animal experiments performed in this study (registration no. LALLF M-V/TSD/7221.3-1.1-054/15). For syngeneic transplantation, we used 129S6/SvEvTac mice, which were bred and maintained in the animal facility of the Rostock University Medical Center, as described previously [14]. Acute myocardial infarction was induced by surgical ligation of the left anterior descending coronary artery (LAD) following thoracotomy. Intramyocardial injections were performed immediately after MI induction, as described before [18]. The MI group received an intramyocardial injection of 10 µL PBS mixed with 10 µL Growth Factor Reduced Matrigel^TM^ Matrix (Corning, Berlin, Germany). The MIC group received a suspension of 1 x 10^6^ CiC in PBS (10µL) mixed with 10 µL matrigel. Injections of 4 x 5 µL were given along the infarct border. The site of injection was visually controlled at the time of transplantation.

The mice were divided into four groups for [^68^Ga]-NODAGA-RGD imaging seven days following MI:

1) SHAM (n = 6)
2) MI (n = 6)
3) MI + blocked (n = 3)
4) MIC (n = 5)

Cardiac function was assessed in separate groups three weeks following MI by cardiac MRI:

5) MI only (n = 6)
6) MIC (n = 6)

### 2.3. PET Imaging 

Mice were anaesthetized by the inhalation of isoflurane (4% for induction and 1–2.5% maintenance during preparation and scanning) for the PET study. We performed all PET/CT scans on a small animal PET/CT scanner (Inveon MM-PET/CT, Siemens Medical Solutions, Knoxville, TN, USA), according a standard protocol. Prior to the scan, the mice were injected intravenously with a dose of approximately 15–20 MBq [^68^Ga]-NODGAGA-RGD via a custom-made micro catheter placed in a tail vein. After an uptake period of 30 min., static images were acquired in prone position for 45 min. The respiration of the mice was controlled and core body temperature was constantly kept at 38° C via a heating pad during the PET scan. Whole body CT scans were acquired for attenuation correction and anatomical land marking. The PET data sets were corrected for random coincidences, dead time, scatter, and attenuation. The reconstruction of the PET images was performed with the three-dimensional (3-D) iterative ordered-subset expectation maximization reconstruction algorithm (3D-OSEM/OP-MAP) with the following parameters: four iterations (OSEM), 32 iterations (MAP), 1.7 mm target resolution, and 128 × 128 matrix size. The data were decay-corrected to the time of injection. Three mice were injected with a blocking dose of cilengitide (0.018 mg/g body weight) 10 minutes prior to the tracer injection in order to verify whether the tracers binds specifically to the RGD sequence [11].

### 2.4. PET Image Analysis 

An Inveon Research Workplace (Siemens, Knoxville, TN, USA) was used for image analysis, as described previously [19]. The PET and CT images were fused by the use of an automated volumetric fusion algorithm and then verified by an experienced reader for perfect alignment. Consecutively, standardized representative volumes of interest (VOI) were manually placed into the infarcted region at the site of maximal focal tracer accumulation. A volume of 20 µL was chosen for the VOI, aiming at covering most the granulation tissue. Correct positioning of the VOI was visually verified in axial, coronal, and sagittal projection. 

### 2.5. Cardiac Magnetic Resonance Imaging and Analysis

**Figure 1:**
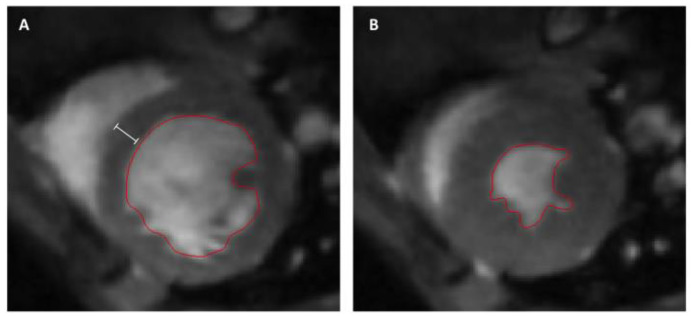
Representative short-axis slices of the left ventricle. The endocardium is marked with a red line for volumetric assessment of the left ventricle. (**A**) end-diastolic image; the white bar represents the interventricular septal thickness (IVSd). (**B**) end-systolic image.

Cardiac magnetic resonance (CMR) measurements were performed on a seven-Tesla small animal MRI system (BioSpec 70/30, maximum gradient strength 440 mT/m, Bruker BioSpin Gmbh, Ettlingen, Germany) equipped with a 1H transmit volume coil (86 mm, volume resonator) and a two-by-two receive-only surface coil array (both Bruker BioSpin GmbH), as described previously [14]. Images for left ventricular ejection fraction (LVEF) measurements were acquired using a IntraGate gradient-echo cine sequences (Intragate Cine-FLASH) in six short-axis planes completely covering the left ventricle following the planning sequences for the axis view. Thereby, the acquisition parameters included: echo time (TE): 2.38 ms, repetition time (TR): 5.89 ms, flip angle: 15°, 14 frames per cardiac cycle, oversampling: 140, averages: 1, field of view (FOV): 29.4 × 25.2 mm, matrix size: 211 × 180, resolution in-plane: 0.14 × 0.14 mm, slice thickness: 1 mm, scan time per slice: 2 minutes (Figure 1). Cardiac function and morphology were assessed from the cine sequences while using the freely available software Segment v2.0 R5165 (http://segment.heiberg.se) [20], as described previously [14].

### 2.6. Histology

The mice were euthanized after the PET scan, hearts were removed, embedded in O.C.T.TM compound (Tissue-Tek^®^; Zoeterwoude, Netherlands), and snap-frozen in liquid nitrogen. For histological examination of the infarction area, the heart tissue was divided into four horizontal levels from the apex to the base. For the discrimination of the scar from uninjured myocardium heart sections (5 μm) were stained with Sirius Red (Division Chroma, Muenster, Germany) and Fast Green FCF (Sigma–Aldrich), respectively. The infarct size and wall thickness were determined by the use of the Axio Vision LE Rel. 4.5 software (Carl Zeiss GmbH).

### 2.7. Tracer Preparation

**Figure 2:**
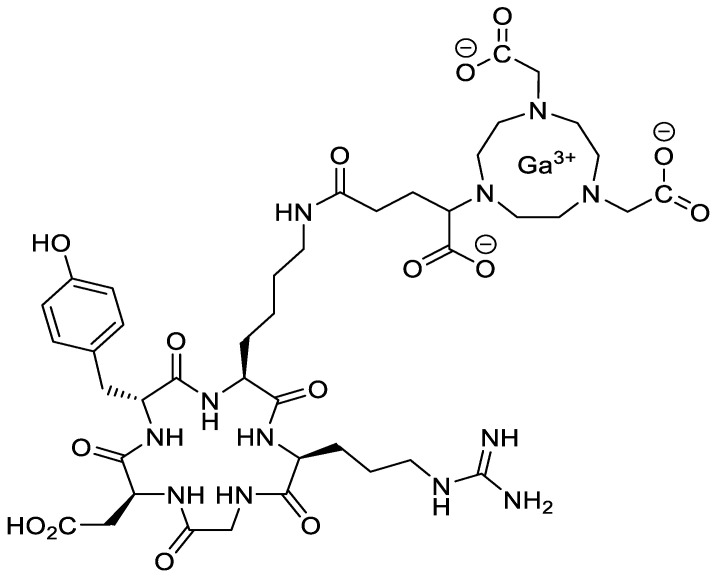
Structure of [^68^Ga]-NODAGA-RGD.

[^68^Ga]-NODAGA-RGD (Figure 2) was synthesized by means of a GRP module (Fa. Scintomics) while using a Ga peptide labeling kit (Sc-01, ABX, Radeberg) and 10 µg of NODAGA-RGD trifluoroacetate (9805, ABX, Radeberg). The sequence is carried out, as follows: the activity from the GalliaPharm generator (Eckert & Ziegler) was trapped on the included PS-H^+^ cartridge, which was eluted into the reactor (preloaded with the NODAGA-RGD dissolved in 3 mL of 1.5 M HEPES) with a sodium chloride solution (1.5 mL, 5 M). The mixture was heated for 10 min. at 125 °C. Afterwards, the solution passed an ethanol-preconditioned Light C18 cartridge, whereby the product was trapped. After rinsing with water, the elution of the product was achieved with 4 mL of a 50% ethanol/water mixture. A 50% ethanol/water mixture replaced the PBS buffer of the kit, which normally fills up the product to an acceptable ethanol concentration. All solvents of the synthesis product were removed via evaporation in order to achieve an activity concentration of about 1 GBq/mL. The dried product was dissolved in 100 µL of a 0.9% sodium chloride solution (Fa. B. BRAUN, Melsungen). The molar activity after this preparation was in the range 20.4–38.1 MBq/nmol. The pH value of the final solution was 6. The activities were determined using a dose calibrator (MED Isomed 2010).

Analytical HPLC was performed using a CS MultoHigh 100 RP18 column (5 µm, 4 × 250 mm) on a Shimadzu HPLC pump and UV detector (220 nm) connected to a radiodetector. Mobile phase: A = water with 0.1% TFA, B = acetonitrile with 0.1% TFA, gradient: 0–0.5 min. 10% B, 0.5–7.0 min. 55% B, 7.0–30.0 min. 55% B. Flow rate 1 mL/min. Radiochemical identity and purity was verified via high performance liquid chromatography comparing the retention time of [^68^Ga]-NODAGA-RGD (t_R_ = 8.4 min.) with those of [^69^Ga]-NODAGA-RGD (9807, ABX, Radeberg, t_R_ = 8.4 min.).

Additionally, thin layer chromatography was performed on a Raytest miniGita Star while using iTLC SG paper (Agilent) and a mixture of NH_4_OAc in water and methanol (1:1) as mobile phase. No free [^68^Ga] could be observed.

### 2.8. Autoradiography

The excised hearts were frozen and embedded in O.C.T.TM Compound (Tissue-Tek^®^). Serial LV short-axis cryosections of 20-μm thickness were obtained. After quick air drying, the sections were exposed to an imaging plate (Dürr NDT Image Plate HR 20x25, Dürr Medical, Germany). Exposure with the samples was carried out overnight due to the limited quantum efficiency of the imaging plates at high energies (such as the 511 keV of PET radionuclides) [21]. Imaging plates were scanned with an image plate scanner (CR 35 BIO, Elysia-raytest GmbH, Germany; internal resolution of 30 μm), and the images were analyzed for background-corrected count densities with an image analysis program (AIDA Image Analyzer, Elysia-raytest GmbH, Straubenhardt, Germany). 

### 2.9. Statistics 

All of the data in this manuscript are presented as mean values ± standard deviation (SD). Student’s t-test was used for statistical analysis of parametric data. The Mann–Whitney test was used for analysis of nonparametric data. Values of *p* < 0.05 were considered to be statistically significant. 

## 3. Results

### 3.1. PET- Imaging of α_v_β_3_-Integrin after Myocardial Infarction

**Figure 3:**
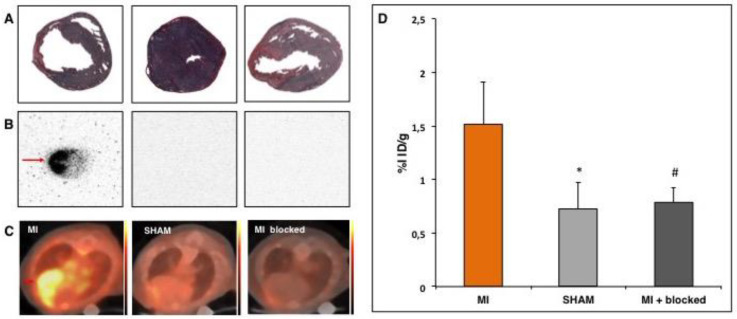
[^68^Ga]-NODAGA-RGD positron emission tomography (PET) seven days after surgical induction of myocardial infarction shows enhanced focal tracer accumulation within the granulation tissue of the infarct scar. (**A**,**B**) Autoradiograms and Sirius Red stainings of adjacent myocardial cross sections. Highest tracer accumulation was detected in the scar area (red arrow), injection of cilengitide 10 minutes prior to tracer application reduced focal activity to SHAM level. (**C**) Axial PET/CT showing high focal uptake of [^68^Ga]-NODAGA-RGD with in the scar, which is suppressed to SHAM level by cilengitide application. Color bars: 0 to 160 kBq/ml (MI) and 0 to 130 kBq (SHAM and myocardial infarction (MI) blocked). (**D**) %ID/g was measured by positioning a VOI of 20µL into the brightest area within the scar. * *p* < 0.005 vs. MI; # *p* < 0.05 vs. MI.

The expression of α_v_β_3_-integrin has been shown in both rats and pigs to be an appropriate surrogate marker for imaging angiogenesis during the healing process of myocardial infarction [10,12]. Yet, a PET tracer for the mouse model is still greatly lacking. Therefore, we assessed [^68^Ga]-NODAGA-RGD for α_v_β_3_-integrin imaging in the mouse model seven days after induction of acute myocardial infarction. The MI animals show significantly higher focal tracer accumulation in the infarct region as compared to the SHAM group (1.51 ± 0.36% ID/g vs. 0.72 ± 0.24% ID/g; *p* < 0.005) (Figure 3D). We injected the competitive inhibitor cilengitide 10 min. prior to the application of the tracer in a subgroup of three animals, which suppressed the uptake of [^68^Ga]-NODAGA-RGD to the level the SHAM group in order to assess the specificity of the tracer (0.79 ± 0.14% ID/g vs. 0.72 ± 0.24% ID/g; *p* = 0.71) (Figure 3D). Tracer accumulation within the granulation tissue of the scar was verified by autoradiography (Figure 3B).

### 3.2. Assessment of Left Ventricular Remodeling Following AMI by the Use of MRI

**Figure 4:**
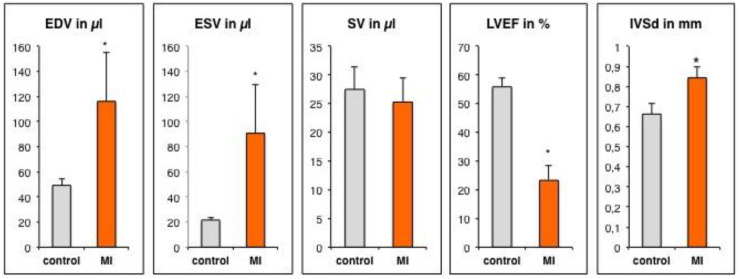
Functional and morphological parameters obtained from cardiac magnetic resonance (CMR) imaging. EDV: enddiastolic volume, ESV: endsystolic volume, SV: stroke volume, LVEF: left ventricular ejection fraction, IVSd: diastolic interventricular wallthickness. Data are presented as mean with SD. * *p* < 0.05 compared to control group. EDV: end-diastolic volume; ESV: end-systolic volume; SV: stroke volume (EDV-ESV); LVEF: left ventricular ejection fraction (EDV-ESV/EDV*100%); IVSd (diastolic interventricular septal thickness).

**Figure 5:**
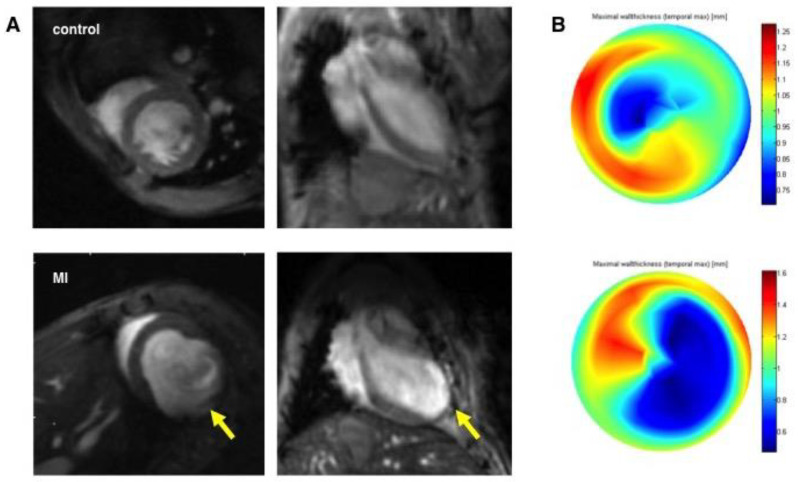
CMR images of representative healthy (upper row) and infarcted animals three weeks after MI (lower row) (**A**) left ventricular (LV) dilation, aneurysm formation in the thinned anteroseptal wall (yellow arrow) and LV hypertrophy can be observed. (**B**) bullseye plots depicting the maximal wall thickness of the left ventricle visualize the thinning of the apex and the anteroseptal wall.

Cardiac morphology and function were measured by the use of magnetic resonance imaging three weeks following myocardial infarction. When compared to healthy mice, the MI group exhibited the distinctive MI induced changes (Figure 4). MI induced extensive apical and anterolateral scar formation with development of apical aneuryms (Figure 5). The dilation of the left ventricle resulted in dramatically increased volume of the left ventricle. EDV (49.2 ± 5.0 µL vs. 116.1 ± 39.0 µL; *p* < 0.005) and ESV (21.7 ± 1.9 µL vs. 90.7 ± 38.7 µL; *p* < 0.005) were both significantly increased. Left ventricular remodeling resulted in a significantly reduced LVEF (55.9 ± 3.1% vs. 23.3 ± 5.3%; *p* < 0.001). Furthermore left ventricular hypertrophy could be detected (IVSd: 0.66 ± 0.05 mm vs. 0.84 ± 0.05 mm; *p* < 0.001) as a compensatory mechanism, resulting in preserved stroke volume (SV: 27.5 ± 4.0 µL vs. 25.3 ± 4.2 µL; *p* = 0.3).

### 3.3. Effect of Cell Therapy on Cardiac Remodeling Following AMI

**Figure 6:**
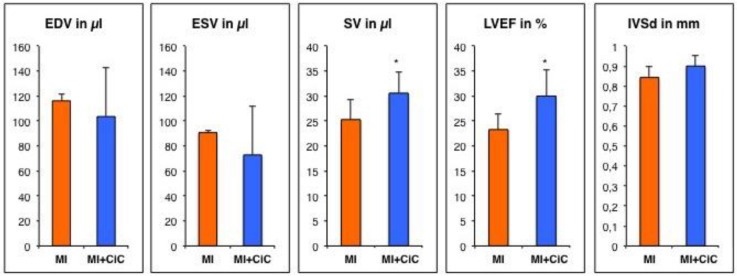
Effect of cardiac induced cells (CiC) transplantation on cardiac function and morphology measured by CMR three weeks after MI induction. Values are mean with SD. * *p* < 0.05 compared to MI group.

Cardiac induced Cells (CiC) were generated, as described previously [14], and injected into the border zone of the infarction after surgical occlusion of the LAD. CMR performed after three weeks showed reduced LV-remodeling resulting in preserved cardiac function as compared to the MI group (Figure 6). Left ventricular dilation was reduced, but it did not reach statistical significance (EDV: 103.6 ± 15.7 µL vs. 116.1 ± 39.0 µL; *p* = 0.44 and ESV: 72.8 ± 15.6 µL vs. 90.7 ± 38.7 µL; *p* = 0.28). CiC therapy resulted in significantly improved functional parameters (LVEF: 30.0 ± 5.0% vs. 23.3 ± 5.3%; *p* < 0.05 and SV: 30.6 ± 3.6 µL vs. 25.3 ± 4.2 µL; *p* < 0.05). Moreover, a trend of more pronounced LV hypertrophy was observed (IVSd: 0.9 ± 0.2 mm vs. 0.84 ± 0.05 mm; *p* = 0.4).

### 3.4. Effect of CiC Transplantation on Angiogenesis and Correlation with LV-Remodelling

We established α_v_β_3_-integrin imaging by the use of [^68^Ga] -NODAGA-RGD PET for detecting the angiogenic response following acute myocardial infarction, as described above. Furthermore, MRI had revealed significant improvement of cardiac function after CiC therapy. We hypothesized that CiC transplantation results in elevated levels of α_v_β_3_-integrin expression within the myocardium, as increased neovessel formation has been shown to reduce cardiac remodeling [22]. 

**Figure 7:**
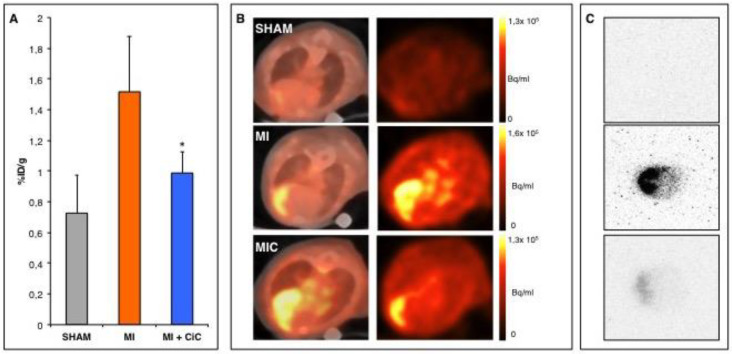
(**A**) Transplantation of CiC significantly reduces uptake of [^68^Ga]-NODAGA-RDG in the infarct region. * *p* < 0.05 compared to the MI group. (**B**) Representative axial images of the respective group: PET/CT fusion images on the left, PET images on the right. Tracer accumulation can be clearly allocated to the infarct region. (**C**) Focal tracer accumulation is located in the infarct region as shown in representative images from autoradiography.

**Figure 8:**
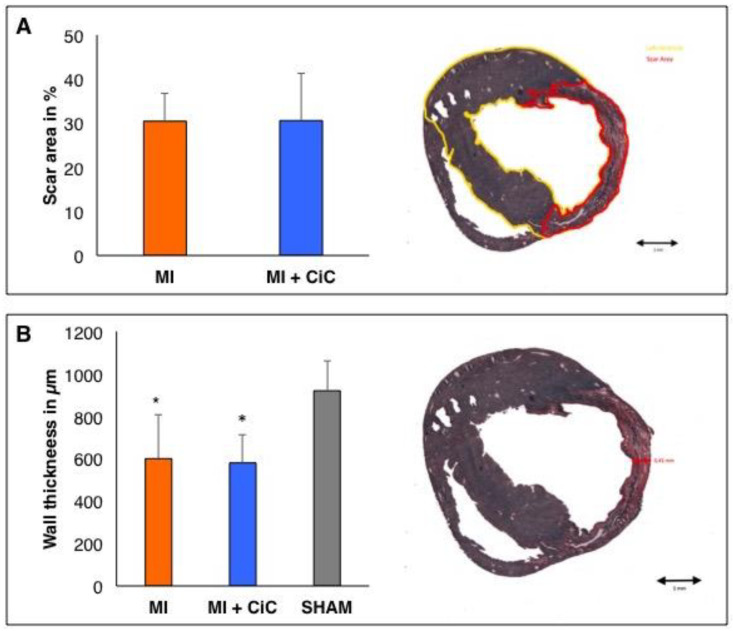
Heart sections of four horizontal infarction levels (5 μm) stained with Sirius Red (collagen) and Fast Green (non-infarcted myocardium). Right side: Representative sections of the MI group showing measurements of (**A**) scar size and (**B**) wall thickness of the infarcted region. * *p* < 0.05 compared to SHAM group.

Yet, intriguingly, [^68^Ga]-NODAGA-RGD accumulation within the infarct zone was significantly reduced by CiC transplantation when compared to the MI group (1.5 ± 0.4% ID/g vs. 1.0 ± 0.1% ID/g; *p* < 0.05; Figure 7). We wondered whether the observed α_v_β_3_-integrin expression correlates with differences in scar size or wall thickness. Yet, we did not find any difference between MIC and MI group based on histological findings (Figure 8). In a previous study, we examined the effect of CiC transplantation on the myocardial inflammation five days after infarct induction [14]. CiC transplantation lead to distinct changes of the inflammatory profile on a cellular level, suggesting changes in the dynamics of infarct healing.

## 4. Discussion

Our study is the first to image angiogenesis based on a α_v_β_3_-integrin targeted PET tracer in a mouse model of acute myocardial infarction. The α_v_β_3_-integrin is highly expressed at the tips of capillaries sprouting into the provisional matrix of granulation tissue [8,23]. Therefore, it represents the most commonly used molecular target in the development of PET tracers for imaging angiogenesis [24]. [^18^F]-Galacto-RGD was introduced in 2001 by Haubner et al. for non-invasive imaging of α_v_β_3_-integrin expression in tumor mouse models [25], and it was rapidly translated into clinical evaluation in cancer patients [26]. Moreover, it was demonstrated that [^18^F]-Galacto-RGD imaging is sensitive enough to visualize α_v_β_3_-expression resulting exclusively from the tumor vasculature [26]. Hence, it was studied to monitor α_v_β_3_-integrin expression following acute myocardial infarction in a rat model [27]. The accumulation of [^18^18]-Galacto-RGD within the infarcted area peaked seven days post MI, correlated strongly with newly formed vessels and it could be blocked by a competitive inhibitor, which suggests the successful imaging of angiogenesis in the process of myocardial healing [27]. Various independent research groups were able to validate the concept of α_v_β_3_-integrin imaging—with both SPECT and PET tracers—as a surrogate marker for angiogenesis in the healing process of ischemically damaged myocardium [13,28,29] and skeletal muscle [30,31].

The multi-step synthesis of [^18^F]-Galacto-RGD is time consuming and it requires an on-site cyclotron, making an automated production process extremely difficult [11,24]. The search for alternative RGD compounds resulted in the development of [^68^Ga]-NODAGA-RGD, which has similar characteristics to [^18^F]-Galacto-RGD, making it an ideal candidate for small animal applications [11,24]. [^68^Ga] tracers have the advantage of easy and fast production with a generator-produced radionuclide [24]. Furthermore, both precursor and the non-radioactive standard are commercially available in cGMP quality [32], endowing [^68^Ga]-NODAGA-RGD with optimal features for potential clinical translation. 

We aimed at establishing [^68^Ga]-NODAGA-RGD based imaging of angiogenesis in a murine model of myocardial infarction against the background that imaging α_v_β_3_-integrin expression following myocardial infarction has already been evaluated in rats. Based on findings from Murry´s group, that formation of granulation tissue and the vascularization of the wound peak seven days after surgical LAD occlusion in mice [33], we chose this time point for [^68^Ga]-NODAGA-RGD imaging. We detected significantly increased tracer accumulation in the infarct area seven days after surgical induction of MI when compared to SHAM operated animals. The exact localization within granulation tissue of the infarct could be verified by autoradiography. The specific binding of [^68^Ga]-NODAGA-RGD to α_v_β_3_-integrin was shown by blocking tracer accumulation down to SHAM levels with Cilengitide. These findings are well in line with previous SPECT studies in the mouse model, showing intense angiogenesis in the infarct region by the use of α_v_β_3_-integrin targeted tracers - [^111^In]-DTPA-cNGR [9] and [^99m^Tc]-NC100692 [34] seven days after surgical LAD occlusion. Against this large body of evidence, we conclude that angiogenesis following MI can be visualized and measured by [^68^Ga]-NODAGA-RGD PET in mice.

We then intended to test the hypothesis that [^68^Ga]-NODAGA-RGD PET might be a valid tool for predicting cardiac remodeling following acute myocardial infarction, as stated by Schwaiger´s group [12]. Therefore, murine ES-derived cardiac induced cells (CiC) were transplanted following MI induction and CMR measured the effect on cardiac remodeling. We chose this cell preparation based on its ability to improve pump function when transplanted after acute myocardial infarction, as previously described by our group [14]. CiC transplantation induced a pronounced shift in the distribution pattern of inflammatory cells within the heart. Thereby, the amount of monocytes was decreased in favor of an increase of M1 and M2 macrophages in both the remote and infarct area of the myocardium. As macrophages play a central role in the process of myocardial healing and ventricular remodeling [35], we hypothesized that angiogenesis—an integral part of wound healing—would be significantly altered by CiC transplantation.

Cardiac remodeling was significantly reduced by CiC transplantation, resulting in significantly improved LVEF and SV, as measured by CMR three weeks following MI induction. The magnitude of LVEF improvement was in the expected range when compared to results from a meta-analysis performed by our group [36]. 

We measured [^68^Ga]-NODAGA-RGD accumulation in the infarct area following CiC transplantation seven days post MI in order to evaluate the correlation between α_v_β_3_-integrin expression and LV-remodeling. Surprisingly, cell therapy significantly reduced focal tracer accumulation in the infarct area. CMR showed reduced LV dilation, resulting in significantly increased stroke volume and LVEF in the cell therapy group. Hence, we reasoned that lower α_v_β_3_-integrin expression in the infarct area might be a consequence of a mere reduction of myocardial damage caused by cell therapy. Yet, scar size and wall thickness of the infarct showed no difference between control and therapy group seven days post MI. The angiogenic response following MI and its therapeutic modulation is probably more complex than assumed by others and us. CiC transplantation significantly alters the cellular inflammatory response profile five days following acute MI, including a marked shift in monocyte and macrophage subpopulations, which play an important role in angiogenesis [37], as recently shown by our group [14]. CiC therapy seems to modify the complex process of myocardial healing, which leads to distinct changes of both inflammatory and angiogenic responses when compared on day five and day seven post MI. In the specific model used by us, both parameters are valid predictors for therapeutic efficacy of our cell therapy. 

Yet, these findings and methods cannot be directly transferred to other mammals or therapeutic approaches targeting myocardial healing, as exemplified in the following paragraph. 

Huang at al. transplanted cell aggregates consisting of human umbilical vein endothelial cells (HUVECs) and cord-blood mesenchymal stem cells (cbMSCs) into the peri-infarct zones in a rat model aiming at angiogenesis induction. Angiogenesis, as assessed by [^68^Ga]-RDG PET, was significantly increased by cell transplantation five days post MI and associated with less cardiac remodeling and a better pump function [13]. They ascribe the increase of angiogenesis on day five to therapeutically induced angiogenesis, which then leads to recovery in blood perfusion.

Lindsey et al. examined the role of the extracellular matrix formation on angiogenesis [34]. Thereby, MMP-9 deletion improved left ventricular function following acute myocardial infarction in mice after seven days. This improvement was associated with an increase of α_v_β_3_-integrin expression measured by NC100692 SPECT seven days post MI. Yet, the infarct size showed no significant difference on day seven, which is well in line with our findings.

Sherif et al. correlated heart morphology and angiogenesis of individual data sets of rats [12]. LV remodeling assessed 12 weeks after acute MI by CMR was associated with lower levels of α_v_β_3_-integrin expression measured by [^18^F]-galacto-RGD PET 7 days post MI. 

A small clinical trial performed [^18^F]-fluciclatide PET in Patients 14±7 days after presentation with acute ST-segment elevation MI. Cardiac function was assessed by CMR within seven days after PET. Tracer accumulation was not correlated with infarct size, but with functional recovery, suggesting that [^18^F]-fluciclatide uptake is not a surrogate of infarction, but relates more to the tissue-healing response to injury [38]. 

The results from these studies seem to be relatively inconclusive. Yet, these data sets and especially the respective conclusions must be carefully interpreted, as the speed of myocardial healing differs considerably between species [33]. Hence, it is of utmost importance to understand what is actually being imaged via RGD-targeted tracers in the respective context. LAD ligation induces a massive wound, which is consecutively replaced by granulation tissue. In mice, the number of newly formed vessels in the infarct peaks seven days post MI, suggesting that α_v_β_3_-integrin expression assessed by PET imaging reflects most of all capillaries sprouting in the freshly formed granulation tissue. 

Murry´s group hypothesized that interventions that accelerate the formation of granulation tissue will reduce ventricular remodeling and, thus, decrease heart failure [33]. Vandervelde et al. compared the healing kinetics of infarcts caused by permanent versus transient occlusion of the LAD in mice [39]. Thereby, LV remodeling and the resulting LVEF impairment was significantly more severe in the permanent occlusion group and furthermore associated with prolonged myocardial healing. Minatoguchi et al. found intra-venous G-CSF application in a rabbit model of myocardial infarction to accelerate the healing process resulting in improved cardiac function and post MI LV—remodeling [40].

Hence, we think that therapies targeting the healing process after acute myocardial infarction are very likely to induce a shift in the speed of myocardial healing, which is specific for the respective therapeutic agent applied. Angiogenesis in the context of granulation tissue formation might peak earlier as a consequence. Therefore, comparisons of different therapeutic applications at the identical time point might be difficult, even in the same species. There are only few studies addressing the speed of healing as a predictor of myocardial regeneration, which suggests that further research in this field is of utmost importance.

## Abbreviations

LAD	left anterior descending coronary artery
MI	myocardial infarction
CiC	cardiac induced cells
mESCs	murine embryonic stem cells
EB	embryoid bodies
LVEF	left ventricular ejection fraction
LV	left ventricle
PET	positron emission tomography
SPECT	single photon emission tomography
CMR	Cardiac magnetic resonance
CT	computer tomography
VOI	volumes of interest
IVSd	diastolic interventricular septal thickness
SD	standard deviation
EDV	end-diastolic volume
ESV	end-systolic volume
SV	stroke volume
